# The development of a decision making pathway for the physiotherapy treatment of adult scoliosis

**DOI:** 10.1186/1748-7161-9-S1-O47

**Published:** 2014-12-04

**Authors:** Tony Betts

**Affiliations:** 1 Royal National Orthopaedic Hospital, London, United Kingdom

## Background

The treatment of adults with pain from scoliosis is a complex and difficult task. The variety of structural pathologies with an adult de-novo degenerative conditions, include stenosis, joint subluxation,and disc degeneration. Mechanical pain is often associated with mal-alignment and deviations from the physiological neutral spine posture/structure. The Side Shift approach has been used at the RNOH to treat Adolescent Scoliosis and Adults in pain. In order to optimize the management of adult scoliosis a decision making pathway was developed. The purpose of the pathway was to inform the clinicians, guide the management and protect the adult patients .Ultimately it was hoped that it would improve patient outcomes.

## Aim

To develop and pilot a decision making pathway which would be robust, measurable, easy to follow and effective.

## Method

Initially a Brain storming session was used to develop the key pathways to assessment and treatment.

Adult patients were given open questions on the goals and expectations of treatment.

patients were shown the pathway and asked for comments.

The pathways was taught to the Hospital department and integrated to the Care pathway file for the treatment of scoliosis.

## Results

A descriptive pathways has been developed and will be presented. Early interim results of outcome and experience to use will be presented.

## Conclusions

A pathway provides a structural decision making process which can aid the correct application of treatment techniques to a condition. This needs to be developed initially by discussion and consensus. Robust and varied analysis is needed to prove its effectiveness and worth, to improving the patient experience.
